# Acute brain responses to hypoglycaemia and hyperglycaemia in adolescents with type 1 diabetes

**DOI:** 10.1007/s00125-025-06548-7

**Published:** 2025-10-07

**Authors:** Michele A. O’Connell, Richard Beare, Betty Messazos, Elisabeth A. Northam, Myles Clarkson Fletcher, Marc L. Seal, Fergus J. Cameron

**Affiliations:** 1 Royal Children’s Hospital Melbourne, Parkville, VIC Australia; 2 Murdoch Children’s Research Institute, Parkville, VIC Australia; 3 The University of Melbourne, Parkville, VIC Australia; 4 National Centre for Healthy Ageing and Peninsula Clinical School, Monash University, Clayton, VIC Australia

**Keywords:** Brain, Brain metabolism, Cerebral blood flow, Functional MRI, Hyperglycaemia, Hypoglycaemia, Insulin clamp, Paediatrics, Type 1 diabetes

## Abstract

**Aims/hypothesis:**

The physiological basis of the well-described neurocognitive decrements and structural brain changes in type 1 diabetes is unclear. We aimed to assess differences in cerebral blood flow (CBF) and neural activity before, during and after induced hypoglycaemia and hyperglycaemia in adolescents with type 1 diabetes.

**Methods:**

An observational hyperinsulinaemic clamp and functional MRI study was conducted. Parallel study arms assessed participants during three consecutive glycaemic phases: baseline euglycaemia (5.0±0.5 mmol/l), either hypoglycaemia (2.6±0.5 mmol/l) or hyperglycaemia (18–20 mmol/l), and euglycaemic recovery (5.0±0.5 mmol/l). During each glycaemic phase, CBF/brain perfusion was measured with arterial spin labelling and brain neural activity was measured with fractional amplitude of low frequency fluctuations. Comparative analyses were based on the seven regional functional parcellation areas (networks) of the cerebral cortex. A Bayesian multi-level regression model was employed to test regional differences in CBF and brain neural activity between the various glycaemic conditions.

**Results:**

Twenty adolescents with type 1 diabetes participated: ten in each of the hypoglycaemic and hyperglycaemic study arms. Relative to baseline, acute hypoglycaemia was associated with substantially reduced brain neural activity (six of seven functional networks); no significant differences in CBF were evident. By contrast, acute hyperglycaemia was associated with widespread increases in brain activity (five of seven functional networks) and decreased perfusion (six of seven functional networks). Hypoglycaemia and hyperglycaemia had symmetrically opposite effects on brain neural activity in the visual, ventral attention, dorsal attention, frontoparietal and default networks. Recovery from both hypoglycaemia and hyperglycaemia was associated with persistent alterations in both brain perfusion and neural activity, relative to baseline, despite >45 min of sustained euglycaemia.

**Conclusions/interpretation:**

Across widespread areas of the brain, both brain perfusion and neural metabolic activity are altered by acute hypoglycaemia and hyperglycaemia in adolescents with type 1 diabetes. Recovery from glycaemic extremes is delayed. These findings offer important further insights into the acute cerebral responses to abnormal blood glucose levels.

**Graphical Abstract:**

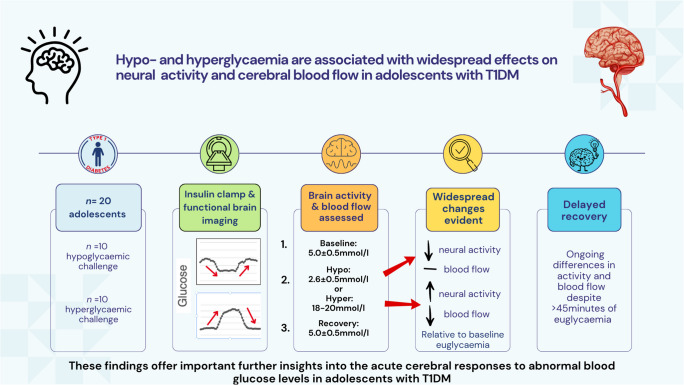

**Supplementary Information:**

The online version of this article (10.1007/s00125-025-06548-7) contains peer-reviewed but unedited supplementary material.


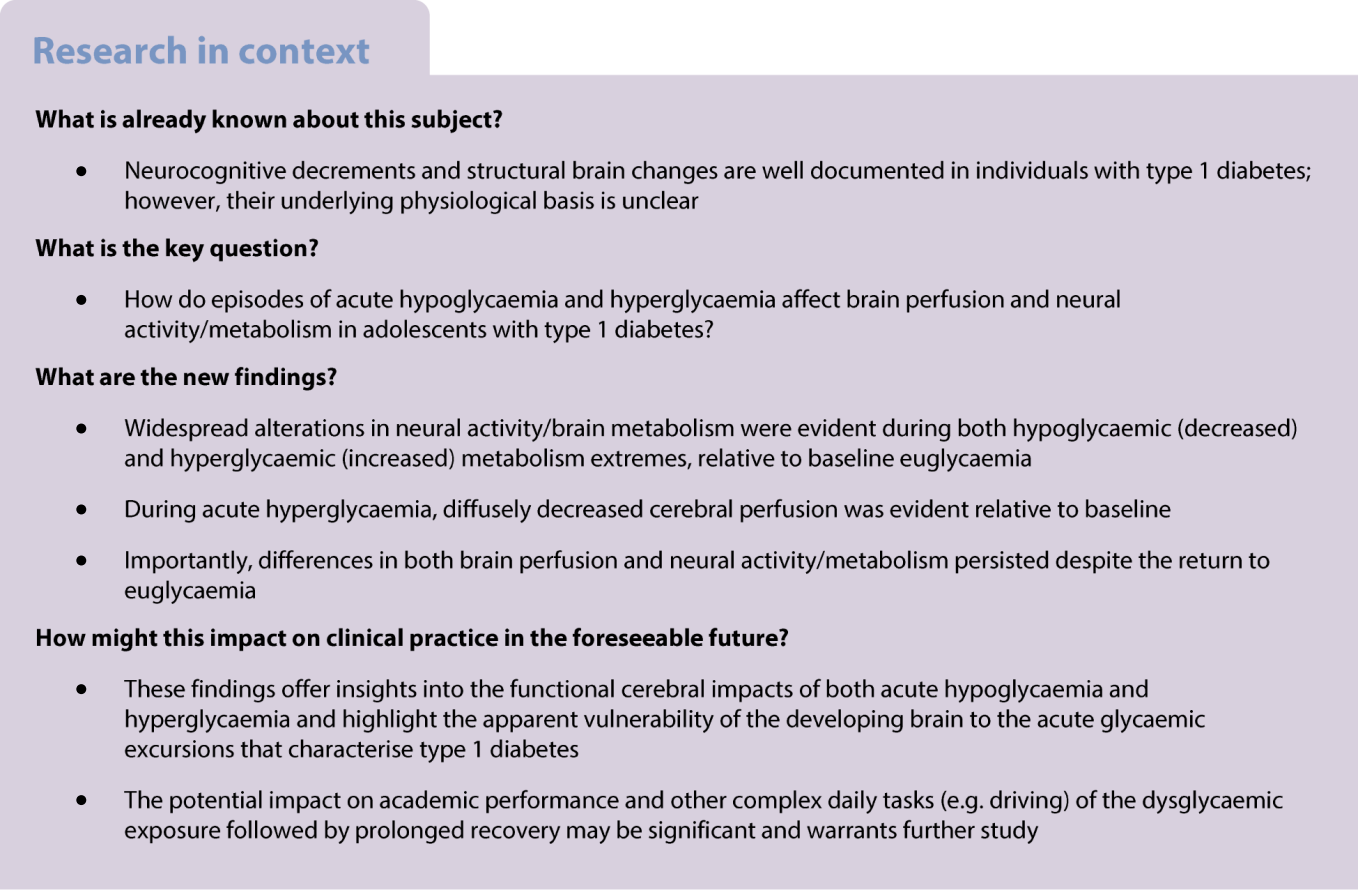


## Introduction

The vulnerability of the brain in type 1 diabetes is well described [1–3]. The neurocognitive skills most affected include working memory, attention, complex information processing and cognitive flexibility [4, 5]. Greatest sequelae are seen in the developing brains of children and adolescents, where clinically significant decrements have been shown to develop over time [6].

Reductions in both total and regional grey and white matter volumes are also well documented in youth with type 1 diabetes [7–12]. A study of children with early age-of-onset diabetes confirmed the evolution of structural brain changes over time and a negative correlation between glucose levels and grey and white matter volumes on longitudinal assessments [13]. While associations between changes in brain volumes and prior exposure to both hyperglycaemia [6, 7, 14, 15] and severe hypoglycaemia [6–8, 16] have been described, the neurobiological mechanisms that underlie these structural and functional changes are unclear.

Since glucose is the preferred substrate for brain metabolism [17], the frequent alterations in circulating glucose that typify type 1 diabetes are likely to play a role. Diabetes-related dysglycaemia commonly encompasses acute and recurrent hypoglycaemia, hyperglycaemia and glycaemic fluctuation. Composite exposure to all of these states across different developmental stages compounds the difficulty in exploring the hierarchy and/or potential synergy of such insults [18].

The concomitant use of insulin clamp techniques with functional MRI (fMRI) facilitates the study of brain function during experimentally manipulated glycaemic phases. Arterial spin labelling (ASL) is a well-validated MRI tool that uses magnetically labelled water proton molecules from arterial blood as an endogenous tracer to provide a non-invasive measurement of cerebral blood flow (CBF). ASL provides comparable CBF results to the gold-standard of positron emission tomography during insulin-induced hypoglycaemia [19]. Fractional amplitude of low frequency fluctuations (fALFF) is a similarly validated non-invasive fMRI technique that measures spontaneous neural activity [20] and correlates strongly with spontaneous brain metabolic activity and blood flow [21].

The aims of this study were to assess differences in CBF and neural activity before, during and after acute hypoglycaemia and hyperglycaemia in adolescents with type 1 diabetes by using a repeated measures multimodal assessment comprising both ASL and fALFF measures.

## Methods

This study was conducted at The Royal Children’s Hospital, Melbourne, Australia; institutional Human Research Ethics Committee approval was received (HREC 32202). Participants were aged 12–18 years with type 1 diabetes >1 year and HbA_1c_ <75 mmol/mol (9.0%). Written informed consent (and personal assent for those <18 years) was obtained for all participants. Exclusion criteria included an IQ <70; a history of psychosis, psychotropic medication or substance abuse; a contraindication to MRI; and a history of neurological trauma or disease, diabetic ketoacidosis, loss of consciousness or seizure associated with hypoglycaemia. A self-monitored capillary blood glucose level of ≤3.9 mmol/l in the 24 h prior to the study day stipulated postponement of assessments.

**Fig. 1:**
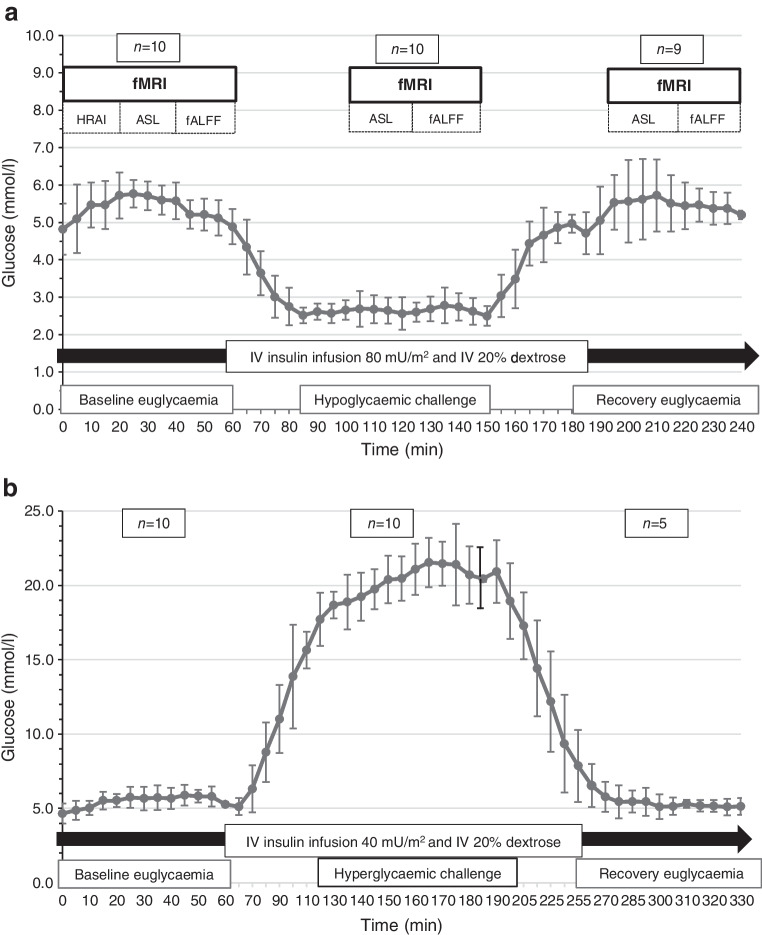
Experimental protocol and mean (SD) glucose levels attained during each study phase of (**a**) the hypoglycaemia challenge study arm and (**b**) the hyperglycaemia challenge study arm. The participants (*n*) who completed a given study phase are shown at the top of each figure. Imaging datasets appropriate for analyses comprised *n*=9 and *n*=10 at baseline, *n*=9 and *n*=9 during the challenge state and *n*=8 and *n*=3 participants at recovery in the hypoglycaemia and hyperglycaemia study arms, respectively (see text for details). HRAI, high-resolution anatomical imaging

The study comprised two parallel arms to assess the acute impact of both hypoglycaemia (2.6±0.5 mmol/l) and hyperglycaemia (18–20 mmol/l) on brain perfusion and activity. As the study design was novel and exploratory, data to inform a power calculation were not available; therefore, the sample size was based on feasibility, with the inclusion of ten participants planned for each glycaemic challenge arm. Participants had neuroimaging assessments at three timepoints – baseline, challenge phase and recovery (Fig. 1). Euglycaemia was identical at baseline and recovery (5.0±0.5 mmol/l).

Outcomes of interest included differences in regional CBF (ASL) and metabolic activity (fALFF) during: (1) each of hypoglycaemia and hyperglycaemia as compared with their respective euglycaemic baseline, (2) euglycaemic recovery as compared with the hypoglycaemic and hyperglycaemic challenge states, and (3) euglycaemic recovery after each of the hypoglycaemic and hyperglycaemic challenge states and baseline euglycaemia.

### Insulin clamp

Participants arrived at 08:00 h, having fasted from midnight. Two intravenous cannulae were inserted, one for insulin and 20% dextrose infusions and one in a dorsal hand vein. A heated hand box (~60°C) was applied to facilitate arterialisation of venous blood for sampling; topical heat packs were used when participants were in the MRI scanner. A primed infusion of rapid-acting insulin aspart was commenced and continued at a constant rate of 80 mU m^–2^ min^–1^ and 40 mU m^–2^ min^–1^ in the hypoglycaemic and hyperglycaemic challenge study arms, respectively. Blood glucose levels were assessed every 5–10 min using a glucose oxidase technique (Yellow Springs Instruments 2300, Yellow Springs, OH, USA). Infusions were run through 12 m lines to allow pumps to be located out of the magnetic field in an adjacent room. Infusion lines and blood samples were passed through purpose-built access points in the wall between these areas.

After clamp initiation, euglycaemia (5.0±0.5 mmol/l) was established and then maintained over 1 h; time zero was then assigned and baseline investigations (below and Fig. 1) were performed. Dextrose infusion rates were then manipulated to attain the glycaemic challenge state before euglycaemia was re-established at recovery. In the glycaemic challenge phase and recovery, fMRI commenced once glucose levels had been in the assigned target range for 15 min. Adrenaline (epinephrine) and noradrenaline (norepinephrine) levels were measured intermittently across the hypoglycaemic challenge arm.

### Neuroimaging acquisition

Neuroimaging was performed using a dedicated clinical research scanner (3 Tesla TIM Trio Siemens MRI scanner; Erlangen, Germany). The MR protocol was designed to maximise the opportunity of capturing changes in brain perfusion and activation across glycaemic phases. High-resolution structural sequences were included at baseline to transform MR images into a common coordinate system for further functional and diffusion image analyses. The following sequences were acquired: (1) High-resolution anatomical images; T1-weighted image, 176 contiguous sagittal slices; 1.0 mm^3^ isotropic voxels; repetition time=1900 ms; echo time=2.15 ms, field of view=256 mm, flip angle=9°; (2) Cerebral perfusion; pulsed ASL (pASL), 24 contiguous axial slices; slice thickness=5 mm, repetition time=3000 ms, echo time=20 ms, 0.7 s label time, 1 s post-labelling delay, flip angle=90°. A total of 100 pASL-MRI frames (50 pASL pairs) were collected per participant. A proton density weighted calibration image (TR=2.5 s, mode=voxelwise) was acquired to allow estimation of perfusion in absolute units (ml [100 g]^–1^ min^–1^); (3) fMRI echo planar (fMRI EPI) images were acquired; repetition time=2400 ms, echo time=40 ms, 3.3 mm^3^ isotropic voxels, 36 contiguous slices providing whole brain coverage. Images for pASL and fMRI outcomes reported herein were acquired in the resting state (rs), hereafter referred to as rs-fMRI.

### MRI processing

Resting state MR data were pre-processed using fMRIPrep to correct distortion and reduce noise and perform quality control [22]. Motion correction was performed using ICA-AROMA (Independent Component Analysis with Automatic Removal of Motion Artefacts) combined with nuisance regression of the mean cerebrospinal fluid signal to remove physiological artefacts [23]. A recent study has identified motion correction approaches based on ICA-AROMA as being among the best options, providing good performance while minimising data loss [24]. fALFF scores, with a low frequency range of 0.01–0.08 Hz were computed using the DPABI toolbox [25]. All scans were manually inspected, and failed or incomplete scans and those with severe motion artefacts were excluded. ASL data was processed to estimate calibrated CBF using FSL BASIL (Bayesian Inference for Arterial Spin Labelling) [26].

Structural T1-weighted images were processed with FreeSurfer 7.0, and CBF and fALFF images were projected to the cortical surface and regional means computed for the Yeo functional parcellation [27–29]. Analyses were based on the seven regional functional parcellation of the cortex by Yeo et al [27]. Voxels without ASL coverage were omitted from calculation of regional mean CBF and fALFF scores.

### Statistical analysis

A Bayesian multi-level regression model was employed to test regional differences in CBF and fALFF signals between the various glycaemic conditions. Separate models were used for CBF and fALFF in each of the hypoglycaemic and hyperglycaemic challenge study arms. This analysis framework includes all glycaemic conditions and brain regions in a single model and utilises partial pooling to stabilise estimates of coefficients and corresponding confidence intervals, which increases statistical power while eliminating the need to correct for multiple comparisons [30].

We used a set of three Bayesian multi-level models for each glycaemic challenge arm to predict regional mean signals from glycaemic condition and brain region while accounting for the repeated measure design. The first two models estimate CBF and fALFF as a function of region and condition, while the third estimates fALFF as a function for region and condition after adjusting for CBF. The models were fitted using the brms package in R 4.2.0 [31, 32] (formulae are available in ESM Methods). Additionally, we tested whether models including sex as a covariate improved the models by using leave-one-out comparison (ESM Methods).

The statistical approach using a hierarchical model with partial pooling employed here was chosen as it is considered particularly suited to datasets with smaller numbers and allows flexibility with missing data (additional information is provided in ESM Methods). The Bayesian framework produces an evidence ratio that characterises the strength of evidence for or against a hypothesis. The guidelines of Kass and Raftery [33] have previously defined evidence categories to report the strength of these evidence ratios (K value) as follows: ‘substantial’ refers to a K value of ≥3.2 and <10; ‘strong’ relates to K of ≥10 and <100, while K values of ≥100 are ‘decisive’. We report regional differences in outcomes between glycaemic conditions for which there is substantial, strong or decisive supporting evidence according to this framework.

## Results

Twenty adolescents participated (Table 1). Their overall median (range) age was 16.4 (12.9–18.6) years, and duration of diabetes was 7.7 (1.3–16.4) years. Median (range) HbA_1c_ at the time of the study was 52 mmol/mol (6.9%) (40–70 mmol/mol (5.8–8.6%)). All hypoglycaemia challenge arm participants had ‘Clarke questionnaire’ scores ≤3, reflecting intact awareness of hypoglycaemia [34]. Nine of ten participants in the hypoglycaemic arm and all hyperglycaemic arm participants were right-handed. All participants completed baseline and challenge conditions. Nine out of ten participants completed the hypoglycaemic recovery phase while only five out of ten participants completed the hyperglycaemic recovery phase (due to difficulties in maintaining intravenous access, *n*=5). The study protocol, participant numbers completing each phase and mean glucose levels attained during each phase of the study are depicted in Fig. 1. Aside from IV access issues encountered in the hyperglycaemia study arm, the protocol was well tolerated by participants with no clinical symptoms necessitating any deviation from protocol. Measurement of catecholamine response demonstrated a rise in mean plasma adrenaline levels during the hypoglycaemic challenge relative to baseline, with return to baseline levels on resumption of euglycaemia; noradrenaline levels were, however, similar throughout this study arm (ESM Fig. 1).

**Table 1:** Participant characteristics

Characteristic	Total	Hypoglycaemia challenge group	Hyperglycaemia challenge group
Participants, *N*	20	10	10
Sex^a^, M:F	11:9	5:5	6:4
Age at baseline, years	16.4 (12.9–18.6)	16.4 (12.9–18.1)	16.4 (14.9–18.6)
Duration of type 1 diabetes, years	7.7 (1.3−16.4)	8.45 (1.9–16.4)	6.23 (1.3–15.8)
HbA_1c,_ mmol/mol; %	52 (40–70); 6.9 (5.8–8.6)	54 (45–68); 7.1 (6.3–8.4)	51 (40–70); 6.8 (5.8–8.6)

Data are presented as median (range) unless otherwise stated

^a^As registered on hospital medical records

Quality control of ASL and rs-fMRI data resulted in the following additional exclusions due to participant movement: one participant in the hypoglycaemia study arm was excluded from all phases, one participant in the hyperglycaemia arm was excluded from the challenge and recovery phases, while a further participant in the hyperglycaemia group was excluded from the recovery phase.

This resulted in datasets for analyses in the hypoglycaemia and hyperglycaemia study arms respectively of *n*=9 and *n*=10 at baseline, *n*=9 and *n*=9 during challenge state and *n*=8 and *n*=3 participants at recovery. The mean glucose levels of participants whose data were suitable for analyses did not differ meaningfully from those of the total cohort: the largest difference of 0.4 mmol/l was recorded at 10% of timepoints during recovery in each study arm, while most mean differences were ≤0.2 mmol/l.

### Differences in CBF (ASL) during baseline euglycaemia, hypoglycaemia/hyperglycaemia challenge and recovery euglycaemia phases

**Fig. 2:**
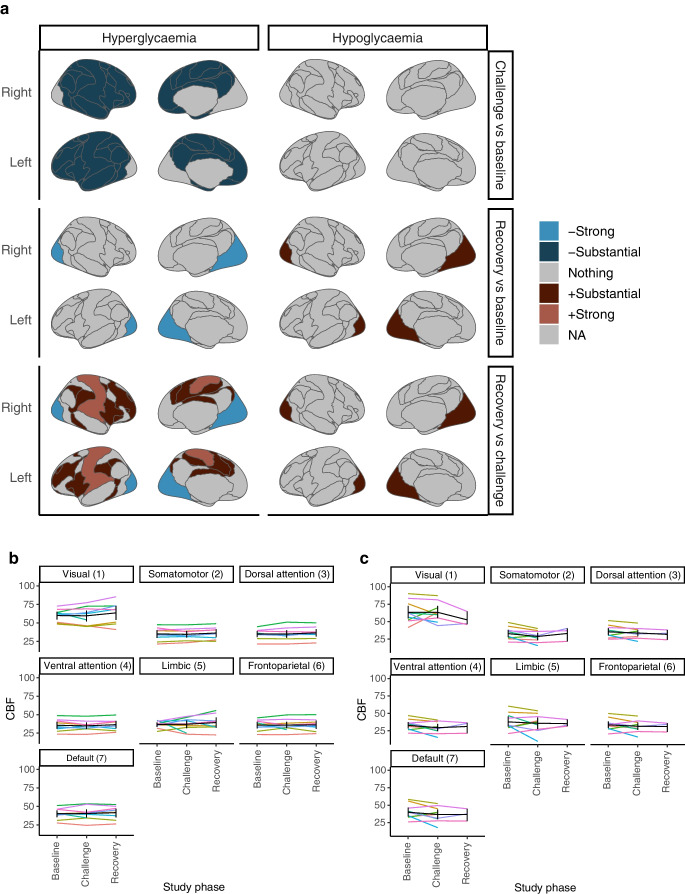
Differences in CBF between study phases (glycaemic states) across the seven functional brain networks. (**a**) Figurative depiction of summated differences in CBF across functional brain networks between the different glycaemic phases of each study arm. Cool colours and warm colours depict reductions (–) and increases (+) in CBF, respectively. (**b**) Trajectory visualisation for regional mean CBF during baseline, challenge and recovery phases in each functional network for the hypoglycaemia challenge arm. Group averaged trajectories and bootstrapped confidence intervals are shown in black; coloured lines depict individual participant trajectories. Analyses included datasets from *n*=9, *n*=9 and *n*=8 participants at baseline, challenge state and recovery, respectively. (**c**) Trajectory visualisation for regional mean CBF during baseline, challenge and recovery phases in each functional network for the hyperglycaemia challenge arm. Group averaged trajectories and bootstrapped confidence intervals are shown in black; coloured lines depict individual participant trajectories. Analyses included datasets from *n*=10, *n*=9 and *n*=3 participants at baseline, challenge state and recovery, respectively

Differences in CBF between study phases across the seven functional brain networks and network areas [27] for both glycaemic challenge arms are demonstrated figuratively in Fig. 2a. Trajectory visualisations for regional mean CBF in the seven functional networks for each glycaemic challenge arm are shown in Fig. 2b (hypoglycaemia) and Fig. 2c (hyperglycaemia). In the hypoglycaemia challenge arm, CBF was similar during acute hypoglycaemia to that observed at baseline; however, perfusion of the visual network was substantially higher during the phase of euglycaemic recovery from hypoglycaemia than during either euglycaemic baseline or the hypoglycaemic challenge phase itself. In the hyperglycaemia study arm, the hyperglycaemic challenge phase was associated with substantially decreased perfusion across six out of seven networks (somatomotor, dorsal attention, ventral attention, limbic, frontoparietal and default networks) compared with that observed at baseline euglycaemia. During the euglycaemic recovery phase following hyperglycaemia, substantial to strong increases in perfusion were evident in the somatomotor, ventral attention and frontoparietal networks compared with that observed during the hyperglycaemic challenge phase itself. However, strongly decreased signals of cerebral perfusion were evident in the visual network during this euglycaemic recovery phase compared with both the hyperglycaemic challenge phase and euglycaemia at baseline (Fig. 2a, c). The changes in perfusion to the visual networks in the recovery phase were symmetrically converse to those seen in the same phases in the hypoglycaemic challenge arm (Fig. 2a).

### Differences in neural activity (fALFF) between baseline euglycaemia, hypoglycaemia/hyperglycaemia challenge and recovery euglycaemia phases

**Fig. 3:**
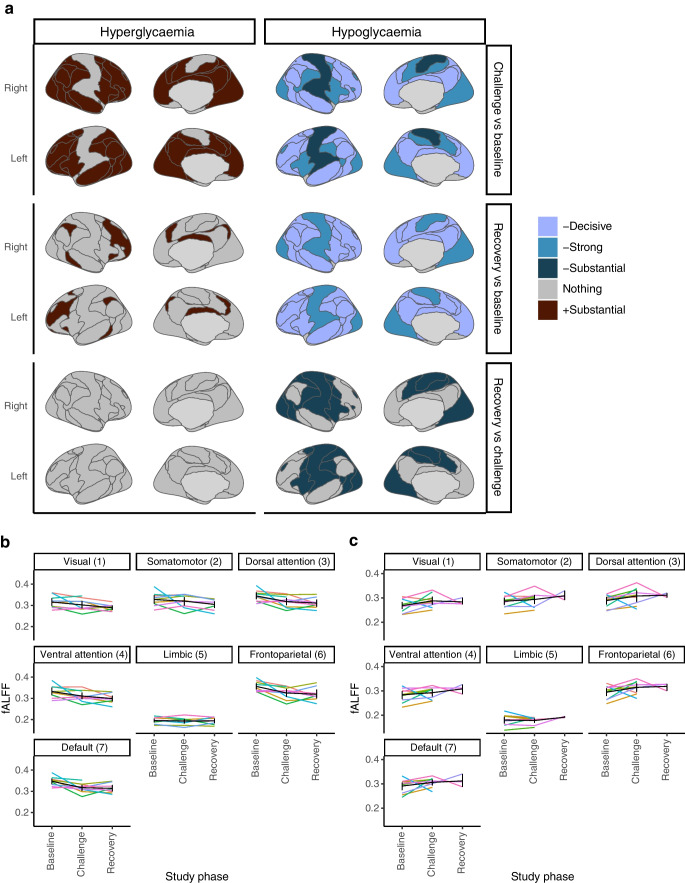
Differences in neural activity (fALFF) between study phases (glycaemic states) across the seven functional brain networks. (**a**) Figurative depiction of summated differences in fALFF-derived neural activity (brain metabolism) across functional brain networks between different glycaemic phases of each study challenge arm. Cool colours and warm colours depict reductions (–) and increases (+) in fALFF, respectively. (**b**) Trajectory visualisation for regional fALFF during baseline, challenge and recovery phases in each functional network in the hypoglycaemia challenge arm. Group averaged trajectories and bootstrapped confidence intervals are shown in black; coloured lines depict individual participant trajectories. Analyses included datasets from *n*=9, *n*=9 and *n*=8 participants at baseline, challenge state and recovery, respectively. (**c**) Trajectory visualisation for regional fALFF during baseline, challenge and recovery phases in each functional network in the hyperglycaemia challenge arm. Group averaged trajectories and bootstrapped confidence intervals are shown in black; coloured lines depict individual participant trajectories. Analyses included datasets from *n*=10, *n*=9 and *n*=3 participants at baseline, challenge state and recovery, respectively

Differences in fALFF across the seven Yeo functional brain networks [27] are shown figuratively for both glycaemic challenge arms in Fig. 3a. Trajectory visualisations for fALFF scores in the seven functional networks for each glycaemic challenge arm are shown in Fig. 3b (hypoglycaemia) and Fig. 3c (hyperglycaemia). The model estimated that adjusted fALFF for ASL changed little after including ASL as a covariate (ESM Methods), so in this instance changes in fALFF appear to equate to changes in metabolic activity.

During the hypoglycaemia challenge phase, there were substantial to decisive decreases in neural metabolic activity in six out of seven networks (visual, somatomotor, dorsal attention, ventral attention, frontoparietal and default networks) compared with those at baseline. Further decreases were evident in the visual, somatomotor, ventral attention and dorsal attention networks during euglycaemic recovery compared with those during the hypoglycaemia challenge phase itself and, overall, strong to decisive reductions in neural activity persisted in the euglycaemic recovery phase in six functional brain networks when compared with those at baseline. By contrast, the hyperglycaemia challenge phase was associated with substantially increased neural metabolic activity in the visual, ventral attention, dorsal attention, frontoparietal and default networks when compared with that at euglycaemic baseline. During recovery from hyperglycaemia, no differences in neural activity were documented relative to the challenge phase itself; however, persistently increased neural activity was evident in the Yeo frontoparietal network relative to that at baseline.

## Discussion

In this study of adolescents with type 1 diabetes, we have shown several novel brain responses to acutely induced hypoglycaemia and hyperglycaemia. First, we have provided substantial evidence that a hyperglycaemic challenge is associated with decreased cerebral perfusion in six out of seven functional brain networks, relative to that observed at baseline euglycaemia. By contrast, an acute hypoglycaemic challenge was not associated with any discernible change in perfusion of any functional brain network. In the recovery phases there were symmetrically opposing ongoing differences in perfusion to the visual networks (increased after hypoglycaemia and decreased after hyperglycaemia challenge phases) relative to baseline. Taken together, these findings indicate that brain perfusion is more readily affected by acute exposure to hyperglycaemia than hypoglycaemia, in terms of both response time and breadth of the CBF alterations noted. Following both glycaemic extremes there are ongoing opposite impacts on visual network perfusion that persist after euglycaemia is restored.

During both the hypoglycaemia and hyperglycaemia challenge phases there were widespread decreases (hypoglycaemia) and increases (hyperglycaemia) in neural activity (fALFF) during the challenge state itself. Again, as with cerebral perfusion, these changes did not return to baseline in the recovery phase. Indeed, widespread differences in neural activity were evident across six of the brain’s seven functional networks during the euglycaemic phase following acute hypoglycaemia. Moreover, we found strong evidence for persistently altered frontoparietal activity following recovery from hyperglycaemia; however, we acknowledge that the small number of participants (*n*=3) with data available for this comparison may impact the broader generalisability of this finding.

A recent study by Deng et al [21] has provided strong evidence that haemodynamic features in the voxel-based blood-oxygen-level-dependent (BOLD) signal at rest are reflective of underlying metabolic demand and support the notion that fALFF can be considered as a metabolic proxy; however, as a voxel-based BOLD signal, both cerebral perfusion and neuronal metabolism contribute to fALFF. Importantly, the multimodal assessment approach (ASL and fALFF) employed here affords us the opportunity to further disentangle the two drivers of fALFF (namely blood flow and neuronal activity) in the context of dysglycaemia. Several of our findings indicate that the changes in fALFF demonstrated here during and in response to acute glycaemic extremes predominantly reflect changes in underlying neuronal activity or metabolism. First, the model estimated that adjusted fALFF changed little after including ASL as a covariate. Second, changes in fALFF were evident in regions where significant differences in ASL were not found, hence these differences in fALFF cannot be explained by alterations in CBF. Finally, in regions where perfusion differences were evident, although differences in fALFF were also found, the latter were in the opposite direction to those that would have been expected if the changes in fALFF signal were predominantly driven by perfusion changes. Indeed, in these areas, the divergent directions of the ASL and fALFF signal changes would in fact make it harder to detect metabolism signals in fALFF, which infers that those reported here are likely to be quite strong.

Altered patterns of regional CBF during experimentally induced hypoglycaemia have previously been reported in adults with diabetes [35, 36]. The analyses reported here extend these findings to a paediatric population. Furthermore, our analyses are novel in their use of a non-invasive multimodal assessment of both CBF and neural activity/metabolism across functional brain networks as opposed to anatomical regions of interest. Although the effects of chronic exposure to hyperglycaemia in type 1 diabetes are increasingly recognised, studies assessing CBF during controlled acute hyperglycaemia in participants with diabetes are limited [37]. Here, we have demonstrated that acute exposure to hyperglycaemia at a level that can be inadvertently encountered by youth with type 1 diabetes in day-to-day life, is associated with widespread changes of reduced blood flow and increased neural activity in a number of brain networks. Adolescents with type 1 diabetes have previously been shown to have significantly reduced spatial working memory capacity and lower frontal cortex metabolites on MR spectroscopy during acute hyperglycaemia when compared with both their own assessments during euglycaemia and those in healthy controls [38]. In addition, areas with dysglycaemia-induced differences in CBF and neural function documented here show overlap with those where acquired volumetric changes in youth with type 1 diabetes have previously been demonstrated [8, 9], underscoring the apparent susceptibility of these regions to dysglycaemia.

There are several key messages that arise from our findings. First, hypoglycaemia and hyperglycaemia seem to have reciprocal impacts on brain metabolism in terms of breadth of functional networks impacted and timing or duration of these effects. Of the two glycaemic extremes examined here, hyperglycaemia had a greater and more rapid impact on cerebral perfusion. Second, the functional brain impacts of hypoglycaemia and hyperglycaemia appear to occur at both a vascular and neural level. During the many different study phases, the changes seen in ASL and fALFF were neither necessarily network-related nor in parallel, implying that in this instance they are not co-dependent outcomes and both CBF and brain metabolism are affected by dysglycaemia. Finally, and importantly, biochemical recovery (resumption of euglycaemia) was not synchronous with either haemodynamic or metabolic recovery.

Our findings offer important further insights into the acute cerebral responses to abnormal blood glucose levels. The neuroimaging modalities employed here are well-validated measures that provide complementary, comprehensive mapping of regional changes in CBF and brain metabolic activity based on change in physiological state (glycaemia). Delayed recovery of cognitive function despite correction of hypoglycaemia has previously been documented in healthy volunteers [39] and our findings lend functional neuroimaging support to this phenomenon in adolescents with type 1 diabetes. There are several potential clinical implications of our findings. Perhaps most notably, despite exposure to euglycaemia for a minimum of 45–75 min (following hypoglycaemic and hyperglycaemic extremes, respectively), dysglycaemic insults continue to affect brain perfusion and metabolism, which may have functional implications for an individual with type 1 diabetes. During this time the brain appears to be operating in a compensated mode. Whether such compensation creates a new ‘vulnerable’ baseline state, rendering individuals susceptible to subtly altered risks of further dysglycaemia or altered responses to dysglycaemia remains to be seen. For example, when acute and opposing dysglycaemia occurs during recovery from a previous event (e.g. rebound hyperglycaemia following inadvertent over-treatment of a hypoglycaemic event, or over-correction of hyperglycaemia resulting in hypoglycaemia) there may be synergistic and ongoing impacts upon brain function that have not previously been recognised.

When taken together with the known clinical decrements in higher order cognitive skills and structural brain changes in youth with type 1 diabetes [6, 8, 9, 38], our findings further underscore the vulnerability of the developing brain to dysglycaemia. Given the ongoing and dynamic nature of dysglycaemia in type 1 diabetes (characterised by repeated glycaemic fluctuations), this remains a difficult mechanism of brain injury to quantify. However, as the potential impact of recurrent episodes of both dysglycaemia and prolonged cerebral recovery on academic performance and other complex daily tasks (e.g. driving) may be significant, the findings we have documented in this sample warrant dedicated future study in larger numbers of young people. The participants in this study were adolescents with good diabetes control; it remains to be seen whether the results differ in younger children at an earlier stage of neurodevelopment or in youth with suboptimal diabetes control. Future larger studies could also explore possible sex-based differences in responses to acute dysglycaemia analyses, which were not undertaken here due to small participant numbers.

Our study has limitations, which include the small sample size and that we did not expose the same participants with diabetes to both glycaemic challenge arms, as this was not considered practical for recruitment purposes. Inclusion of a control group (either adolescents without diabetes or participants with type 1 diabetes assessed at similar timepoints while held in sustained euglycaemia during a hyperinsulinaemic clamp) would also have been beneficial but was not feasible owing to resource limitations. Our study was explorative in nature using novel rs-fMRI outcome variables with a dynamic model of dysglycaemia. Thus, power calculations were not undertaken in an a priori fashion. Additionally, the study protocol itself was rigorous with difficulties in maintaining peripheral intravenous access, which led to reduced numbers completing the hyperglycaemia challenge arm. The complexities of performing an insulin-dextrose clamp while participants were undergoing an MRI meant that deviations from target glucose levels (from the tight pre-defined ranges) during the challenge phases had to be tolerated in the interests of completing the imaging protocol. Mean glucose levels attained at all timepoints were nonetheless well within clinical hypoglycaemic/hyperglycaemic ranges. Furthermore, although the participants had Clark scores indicating hypoglycaemic awareness, it is possible that they encountered unrecognised hypoglycaemia in the day prior to the study that may have impacted the results. Despite every effort, the number of incomplete protocol completions was perhaps a reflection of an arduous study protocol. The hyperglycaemia challenge experienced in the study protocol would not have been feasible to replicate in a control group with intact endogenous insulin secretion. The strengths of this study include the characteristics of our study cohort, the rigorous clamp protocol and the use of a non-invasive multimodal rs-fMRI assessment strategy (overcoming the ethical constraints relating to use of positron emission tomography in a paediatric research study). Our adolescent cohort had a relatively short duration of diabetes with relatively good glycaemic control. None had any evidence of microvascular or macrovascular complications, and all had intact hypoglycaemia awareness. It is thus reasonable to equate our observations of altered perfusion and neuronal metabolism to a primary brain response to acute dysglycaemia.

### Conclusions

In summary, we have demonstrated that across widespread areas of the brain, both brain perfusion and neural metabolic activity are altered by acute hypoglycaemia and hyperglycaemia in adolescents with type 1 diabetes. Moreover, recovery of brain perfusion and neural activity from these glycaemic extremes is delayed. Together, these findings serve to increase our understanding of the neurobiological mechanisms that may underlie the known structural and functional central nervous system changes described in type 1 diabetes.

## Supplementary Information

Below is the link to the electronic supplementary material.

Supplementary file1 (PDF 347 KB)

## Data Availability

Datasets generated from this study are available from the corresponding author on reasonable request.

## Abbreviations

ASL: Arterial spin labelling
BOLD: Blood-oxygen-level-dependent
CBF: Cerebral blood flow
fALFF: Fractional amplitude of low frequency fluctuations
fMRI: Functional MRI
ICA-AROMA: Independent component analysis with automatic removal of motion artefacts
rs-fMRI: Resting state functional MRI
