# Supplementary material for: Acute brain responses to hypoglycaemia and hyperglycaemia in adolescents with type 1 diabetes
Source: Diabetologia. 2025 Oct 7;68(12):2670–81. doi: 10.1007/s00125-025-06548-7 (PMC12594713; doi:10.1007/s00125-025-06548-7)
Supplement: Supplementary file 1 — Supplementary file1 (PDF 347 KB) [file 125_2025_6548_MOESM1_ESM.pdf]

Electronic Supplementary Material (ESM)

ESM Methods

Analyses

Justification for modelling approach used:

The statistical modelling employed, namely partial pooling implemented using Bayesian multilevel models, was selected as this method has a number of strengths that were pertinent to our study design. This approach allowed all regions to be included in a single model which affords better estimation of confidence intervals for each estimate and avoids the need to correct for multiple comparisons.
The "partial pooling" approach seeks a balance between treating each group entirely independently and pooling all data together. Partial pooling approaches represent variables of interest – in this case glycaemic condition (baseline / challenge/recovery) and brain region (based on the 7 regions of Yeo) – as random effects. The hierarchical models embrace the following idea: although each group is unique, having been sampled from the same population, all groups are connected and thus might contain valuable information about one another (i.e. they are expected to bear some degree of similarity instead of being completely unrelated). This statistical approach therefore explicitly models data heterogeneity/similarity in variables of interest.
Bayesian regression performed in this manner has been shown to be particularly beneficial when sample sizes are small, as was the case for our study, as estimates in small-sample-sizes are informed by the population parameters, affording greater precision.

Using hierarchical modelling allowed us to handle participants who drop out at different stages without special treatment. Traditional linear modelling might represent data collected by our study as rows of 3 variables (baseline cbf, challenge cbf, recovery cbf) and one row per participant, with dropout leading to a missing value in the recovery phase. Hierarchical modelling represents the data differently, with one variable indicating the phase (i.e a categorical variable with levels of baseline, challenge and recovery) and a second column with the measure. Each participant who completes the study will have 3 rows. Study dropout will remove a row, but leave other measures associated with that participant intact.
Hierarchical models are therefore well suited to longitudinal studies because they deal naturally with common scenarios, like study dropout.

MRI analyses were performed by customised automated pipelines. The pipelines were created by RB, who was not blinded. However, the same processing pipeline was used for both groups and none of the pipeline parameters were group dependent.

Model formulae

Partial pooling approaches [1] implemented using Bayesian multilevel models, were used, allowing all regions to be included in a single model. Models were fitted using the brms package and R 4.2.0 (more citations). Model formula, in R syntax, had the following structure:

brainMeasure ~ (Condition|Region) + (1|ID) + covariates

where brainMeasure is either the cerebral blood flow or fALFF score for a region, Condition is a factor with levels (baseline, challenge, recovery), Region is a factor with seven levels corresponding to Yeo regions and ID is a participant identifier to account for multiple measures per individual.

Partial pooling approaches represent variables of interest – in this case Condition and Region – as random effects.

<u>Covariate selection</u>

Leave-one-out comparison was used to test CBF models with and without participant sex as a covariate for hypoglycaemic and hyperglycaemic groups. The differences were either small and lower than the standard error (for the hyperglycaemic group, expected log pointwise predictive density (elpd) difference =-0.2, se=0.3) or zero. Thus, the simpler model, without sex, was preferred throughout.

Leave-one-out comparison was also used to test whether the fALFF model was better with a CBF covariate than without. The model without CBF was superior for both hyper and hypoglycaemic groups, although the elpd difference was lower than the standard error estimate for the hypoglycaemic group (hypo: elpd difference = -0.2, se=2.5; hyper: elpd difference = -1.1, se=0.3). The simpler model, without covariates, was thus preferred.

ESM Results

ESM Fig 1 Catecholamine responses – hypoglycaemia challenge arm

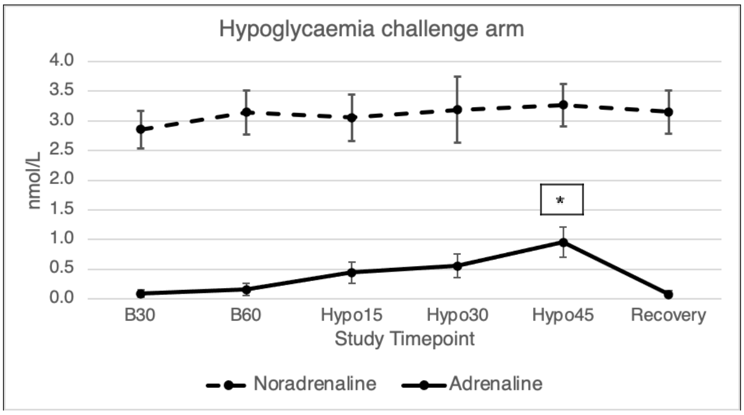

Mean (+/- std error of the mean) catecholamine levels as measured in hypoglycaemia challenge arm participants at various study timepoints.
*p <0.03 for comparison with baseline (both B30 and B60 timepoints)

References:

1. Gelman A, Hill J. Data Analysis Using Regression and Multilevel/Hierarchical Models. Cambridge: Cambridge University Press; 2006.
